# Average Dielectric Property Analysis of Complex Breast Tissue with Microwave Transmission Measurements

**DOI:** 10.3390/s150101199

**Published:** 2015-01-09

**Authors:** John D. Garrett, Elise C. Fear

**Affiliations:** Schulich School of Engineering, University of Calgary, 2500 University Dr. NW, Calgary AB T4N 1N4, Canada; E-Mail: garrettj@ucalgary.ca

**Keywords:** dielectric materials, dielectric measurement, biomedical engineering, biomedical imaging

## Abstract

Prior information about the average dielectric properties of breast tissue can be implemented in microwave breast imaging techniques to improve the results. Rapidly providing this information relies on acquiring a limited number of measurements and processing these measurement with efficient algorithms. Previously, systems were developed to measure the transmission of microwave signals through breast tissue, and simplifications were applied to estimate the average properties. These methods provided reasonable estimates, but they were sensitive to multipath. In this paper, a new technique to analyze the average properties of breast tissues while addressing multipath is presented. Three steps are used to process transmission measurements. First, the effects of multipath were removed. In cases where multipath is present, multiple peaks were observed in the time domain. A Tukey window was used to time-gate a single peak and, therefore, select a single path through the breast. Second, the antenna response was deconvolved from the transmission coefficient to isolate the response from the tissue in the breast interior. The antenna response was determined through simulations. Finally, the complex permittivity was estimated using an iterative approach. This technique was validated using simulated and physical homogeneous breast models and tested with results taken from a recent patient study.

## 1. Introduction

Microwave breast imaging has been proposed to detect and diagnose malignant breast tissue, to monitor breast health and to assess breast density. Prototype and pre-clinical systems have been developed based on microwave tomography [1–4] and radar techniques [5–8]. These imaging methods create 2D or 3D images related to the dielectric properties (DPs) and/or the dielectric contrast of breast tissue. In order to improve microwave breast imaging results, *a priori* information about the average DPs can be used. For radar-based systems, average DPs are used to estimate the average phase velocity through the breast, which is used to focus the beam during image reconstruction. For microwave tomography, average DP information can be used to provide an initial estimate and also to constrain the imaging results.

There are several challenges involved in estimating the average DPs of breast tissue. First, the DPs of biological tissues, including breast tissue, are dispersive with characteristic relaxations [9–11]. The Debye model can be used to approximate these relaxations. For a single-pole relaxation, the Debye model for complex permittivity ϵ^*^) is written as:

(1) $$\in^{*}\left(\omega \right)=\in_{\infty }+\frac{\in_{s}-\in_{\infty }}{1+j\omega \tau }+\frac{\sigma_{s}}{j\omega \in_{0}}$$

where ϵ*_∞_* is the optical permittivity, ϵ*_S_* is the static permittivity, *τ* is the relaxation time, *σ_S_* is the static conductivity, ϵ_0_ is the permittivity of free-space and ω is the angular frequency. Another challenge is that breast tissue is highly heterogeneous. This gives rise to multipath in the breast tissue and interference and resonances in the transmission measurements.

The literature reports many techniques for estimating DPs of bulk materials [12,13], including biological tissues. Methods, such as filled transmission lines or waveguides, require samples to be formed to fit the fixtures. These methods are not suitable for *in vivo* tissue measurements. Open-ended coaxial probes have been used extensively for measurements of excised tissues (e.g., [11]); however, the limited sensing volume does not permit the evaluation of the average properties of larger structures. For evaluating larger samples, free-space transmission measurement techniques have been developed. These systems consist of directive antennas placed on either side of planar samples. The measured reflection and transmission coefficients are used to estimate the dielectric properties, often with time gating of the signal to remove spurious reflections and deconvolution of the antenna response to permit a plane wave model to be employed. In this paper, we discuss a transmission measurement system that adapts ideas related to free-space transmission measurements in order to estimate the average properties of dispersive breast tissues. A key factor in the design of such systems is placing the sensor in contact with the skin in order to avoid dominant reflections from the skin and preferentially couple energy in the breast. To date, limited work has been reported on microwave measurements with ultra-wideband (UWB) sensors in contact with the skin (e.g., [14,15]) and on developing methods to estimate the average DPs from such measurements.

In an effort to estimate the average DPs of breast tissue, a transmission measurement system has been developed at the University of Calgary. The initial system used two ultra-wideband (UWB) antennas to measure the transmission of microwave signals across the breast [14,16]. The antennas were required to contact the breast due to the significant reflection from the skin and attenuation through the breast tissue. Additionally, the breast and the antennas were placed in a bath of saline solution to reduce the effects of external multipath (*i.e.*, energy traveling outside of the breast tissue). The antennas were placed in contact with the skin for the first measurement. Additional measurements were then taken after reducing the separation distance between the sensors (typically by 5, 10 and 15 mm), which lightly compressed the breast tissue. The measured transmission coefficients from two separation distances were then analyzed to estimate the average DPs. This was done by assuming that the only difference between two measurements from two separation distances was due to the change in transmission length through the same average DPs. By subtracting two results, the response due to the added tissue length could be (approximately) isolated. A uniform plane wave model was used to estimate the DPs from the tissue response. This method was successful with low DP breast tissue; however, it was sensitive to multipath. Multipath was characterized by large notches in the transmission coefficient spectrum and caused errors in the average DP estimation.

Recently, a second transmission measurement system was reported [17]. This system incorporates shielded UWB antennas (*i.e.*, “Nahanni sensors”) in contact with the breast [18]. This avoids the requirement for a lossy immersion medium, which is beneficial for patient comfort and the ease of testing. In addition, the shielding permits incorporation of multiple sensors; estimating average DPs at multiple sensor locations provides insight into the property distribution in the breast. Currently, a single average permittivity value is estimated from the transmission measurements using a time-delay spectroscopy approach. It is of interest to provide both average permittivity and conductivity of the tissues over a broad frequency range.

The objective of the work presented in this paper is to develop a new method to analyze transmission coefficients recorded at the shielded sensors in order to provide an accurate average DP estimation. This method must be resistant to multipath and provide a complex permittivity estimate over a wide range of frequencies. The technique will be evaluated through testing with breast phantoms. When applied to patient data, the method must provide reasonable results. This approach uses a different sensor than used in our previous transmission measurement analysis. Moreover, this new method does not require multiple measurements to estimate the average DPs.

In this paper, Section 2 describes the methodology used to collect measurements, to analyze the transmission coefficient and to estimate the average DPs. Section 3 further investigates and validates the method with simulated and measured data collected from test objects. The average DP estimation technique is used with measured data obtained during a recent patient study in Section 4. Section 5 concludes the study.

## 2. Methodology

### 2.1. Measurement

Shielded UWB antennas, dubbed the Nahanni sensors, have been developed to collect microwave signals transmitted through or reflected from biological tissues [18]. These sensors feature a dielectrically-loaded Vivaldi antenna encapsulated in a 25.4-mm diameter cylindrical conductor. They have an operating bandwidth (defined as |*S*_11_| ≤ −10 dB) extending from 1.8 to over 12 GHz. With two antennas aligned aperture-to-aperture and touching, the measured transmission (*S*_21_) ranged from −5 dB at 2 GHz to −20 dB at 12 GHz (*i.e.*, −2.5 dB and −10 dB per antenna, respectively). This loss is primarily due to the dielectric material used to load the antennas.

**Figure 1.**
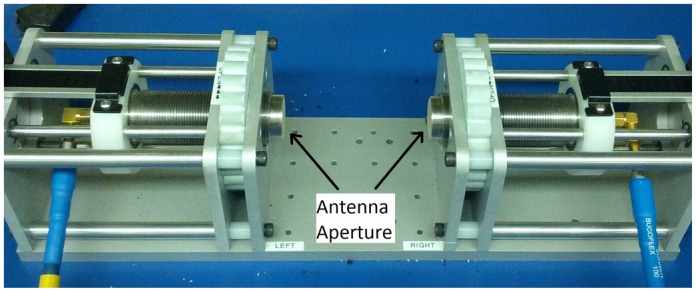
The system used to measure the transmission coefficients of breast phantoms.

Although a 5 × 5-array prototype has been created using the Nahanni sensors [17], a 1 × 1 system was chosen to develop the average DP estimation techniques, due to its simplicity (Figure 1). This system features two Nahanni sensors with apertures aligned and allows the user to control the separation distance with plastic thumb-wheels. The separation distance is read out electronically on two digital calipers (not shown in Figure 1).

Each sensor was connected to a vector network analyzer (VNA, Agilent PNA-L N5232A, Santa Clara, CA, USA) with a 1.5-m coaxial cable. The VNA recorded the scattering-parameters from 50 MHz to 10 GHz at 1601 points. An intermediate frequency bandwidth (IFBW) of 1 kHz and a stimulus power of 0 dBm were used to ensure sensitive results, but the stimulus power was low enough to ensure insignificant tissue heating. Prior to measurement collection, the coaxial cables were disconnected from the UWB sensors, and the system was calibrated with an electronic calibration unit (Agilent N4691A 3.5-mm Electronic Calibration Kit, Santa Clara, CA, USA). After the calibration, the coaxial cables were carefully reconnected to the UWB sensors.

**Figure 2.**
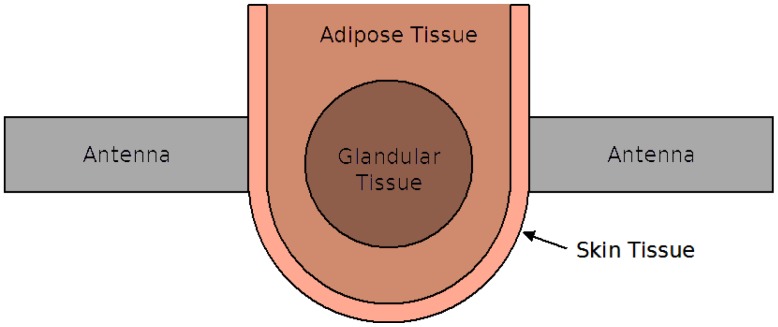
A block diagram of the transmission system, shown in light grey, and a breast model included as the object-under-test.

To measure the s-parameters, the object-under-test (OUT) was placed between the UWB sensors. The sensors were then moved inwards using the thumb-wheels until good contact was achieved across the entire aperture of the antennas. Each measurement took approximately 1.6 s for the VNA to sweep through the frequency range. The overall system can be approximated by the diagram shown in Figure 2. In this case, a breast consisting of adipose, skin and glandular tissue is the OUT.

### 2.2. Signal-Processing

The measured scattering parameters are analyzed to obtain estimates of the average DPs. First, time gating is applied to reduce the impact of multipath. Next, the antenna response is estimated and removed from the data. Finally, the averaged properties are approximated by identifying the parameters of the Debye model that best fit the processed data.

#### 2.2.1. Time-Gating

**Figure 3.**
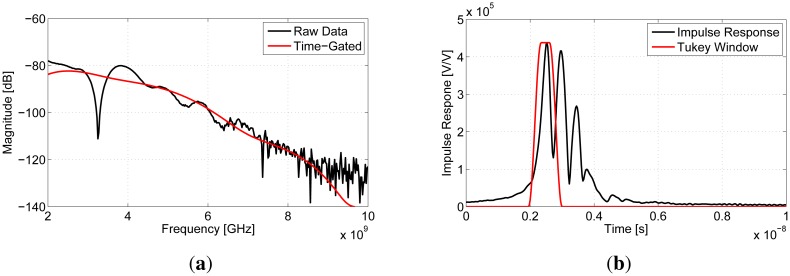
Transmission measurements collected from a patient and exhibiting strong multipath characteristics. (**a**) Frequency domain; (**b**) time domain.

Multipath occurs when the transmitted signal travels along different paths through the breast that likely have different properties. The signals then interfere at the receiving antenna. Since these signals are traveling different distances, multiple peaks in the time domain often occur. An example of a measurement experiencing multipath is shown in Figure 3. These data were taken from a patient study using the initial transmission system (approved by Conjoint Health Ethics Research Board, University of Calgary, Study ID 23244) [14]. In the frequency domain, large notches are seen; in the time domain, multiple dominant peaks are noted. Time-gating can be used to select a single peak in the time domain and isolate a single path. This is a well-known technique that has been used in free-space DP measurement systems to remove reflections from the environment and ringing in the antennas (e.g., [19–21]).

The first step in time-gating was transforming the transmission coefficients from the frequency domain to the time domain with an inverse chirp-z transform (ICZT) [22,23]. The ICZT is beneficial because it allows for the control of the resulting time step and the number of points in the time domain. Likewise, the CZT is useful, because a range of frequencies can be chosen independently from the time step. Next, to isolate components of the signal corresponding to paths between the antennas, peaks in the data were identified and used to create windows. Independent Gaussian curves were fit to the data using a simple optimization algorithm. This allowed for the center time and the width of each peak to be determined. From these Gaussian curves, a single peak was selected for time-gating, and the center frequency and width of this peak were used to create the window. A Tukey window was used, as seen in Figure 3b, because it allowed for the shape of the peak to be preserved while reducing the ringing that would result from a purely rectangular window. The width of the window, taken from the Gaussian curves, allowed for a single peak to be (mostly) isolated, while reducing the influence of other peaks within the window. Since this approach does not require any input from an operator to fit the curves, it removed any operator bias and allowed for repeatable results that were independent of who was applying the technique. Typically, the dominant peak was selected through this process; however, in the case of multipath, where there are multiple dominant peaks, each peak was time-gated and analyzed separately. In Figure 3b, the first peak was chosen. After time-gating, the transmission coefficient was returned to the frequency domain using a CZT. As seen in Figure 3a, time-gating typically removes the large notches experienced in the frequency domain.

#### 2.2.2. Antenna Compensation

The measured transmission coefficient contains the response due to the breast tissue, as well as the response due to the UWB sensors. The antenna response must be removed to isolate the breast tissue data. Typically, free-space DP estimation techniques use a reference measurement (*i.e.*, a measurement with the sample removed) to measure and remove the antenna response [20,21]. This technique does not work for the transmission systems investigated in this paper, because the antennas are placed in contact with the breast tissue. Due to this contact, the antenna response is dependent on the DPs of the OUT.

To describe the transmission system, Friis' transmission equation for free-space can be adapted to account for propagation through a lossy material [24]:

(2) $${\left[S_{21}\left(f,R\right)\right]}^{2}=\frac{P_{RX}\left(f,R\right)}{P_{TX}\left(f,R\right)}=\left(1-{\left|S_{RX}\right|}^{2}\right)\left(1-{\left|S_{RX}\right|}^{2}\right)\frac{G_{RX}G_{TX}}{4\beta^{2}R^{2}}e-2\alpha R_{e}-j2\beta R$$

where *P* is the power measured at each antenna, *G* is the antenna gain, *β* is the phase constant, *α* is the attenuation constant, *R* is the distance between the antennas, *S* is the reflection coefficient at each antenna and the *RX* and *TX* subscripts denote the receiving and transmitting antennas, respectively. Note that the variables *G, β, α* and *S* are all functions of the frequency; however, the frequency dependence was omitted from the equations for brevity. Appendix A examines the assumption of uniform plane wave propagation in Equation (2) for the Nahanni antennas. Since both antennas are identical, we can reduce the antenna response to a single term (1 − |*S_RX_*|^2^) *G_RX_* = (1 − |*S_TX_*|^2^)*G_TX_* = *G*. This gives:

(3) $$S_{21}\left(f,R\right)=\frac{G}{2\beta R}e-\alpha R_{e}-j\beta R$$

where *α* is the attenuation constant. Therefore, if the antenna response (*G*) can be determined, the path loss can be isolated, and the average DPs can be estimated.

**Figure 4.**
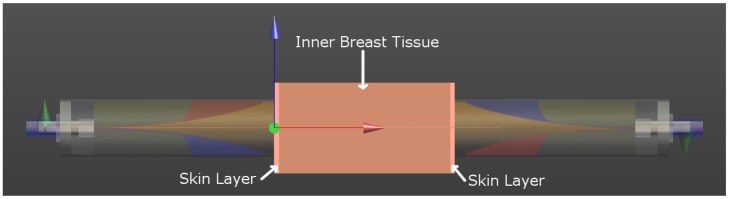
Three-dimensional model used for simulating the transmission system. Two complex Nahanni sensors are present along with a breast model consisting of two 20-mm skin layers and a 56-mm homogeneous interior.

To investigate how the antenna response changes with different materials between the sensors, electromagnetic simulations were created with finite-difference time domain (FDTD) software (SEMCAD, Speag, Zurich CH). The simulation consisted of two complex UWB Nahanni sensor models in contact with a simple breast tissue model, as shown in Figure 4. The breast tissue model was composed of three planar slabs: a 56 mm-thick homogeneous tissue layer representing the interior of the breast (shown in a dark skin color in Figure 4) and two 2 mm-thick layers representing skin on each surface (shown in a lighter skin color). The thickness of the homogeneous tissue layer was based on recent participant results where separation distances were typically between 50 mm and 70 mm. For the skin layer, the thickness was based on results taken from the literature (e.g., [25]).

The excitation pulse had a center frequency of 5.5 GHz and a bandwidth of 9 GHz. The simulation was generated with 14 MCellsand was run for 10 ns. The simulation model is shown in Figure 4.

For the DPs of the skin layers, wet skin results from [10] were used. For the breast tissue, a variety of DPs based on data from [11] were tested. The properties were bounded by fatty tissue results (“Group 3”) and glandular tissue results (“Group 1”). A linear range of 10 tissues was then created between these tissue groups. The DPs of these tissues are dispersive, and the Debye model values are given in Table 1. A simulation was performed with each of these 10 tissues filling the region between the skin layers.

To isolate the antenna response, the theoretical path loss (PL) was found using:

(4) $$PL\left(f,R\right)=\frac{1}{2\beta R}e-\alpha R_{e}-j\beta R$$

This was deconvolved with the simulated transmission coefficient to isolate the antenna response (assuming that Gdoes not depend on the separation between the sensors):

(5) $$G\left(f\right)=\frac{S_{21}\left(f,R\right)}{PL\left(f,R\right)}$$

**Figure 5.**
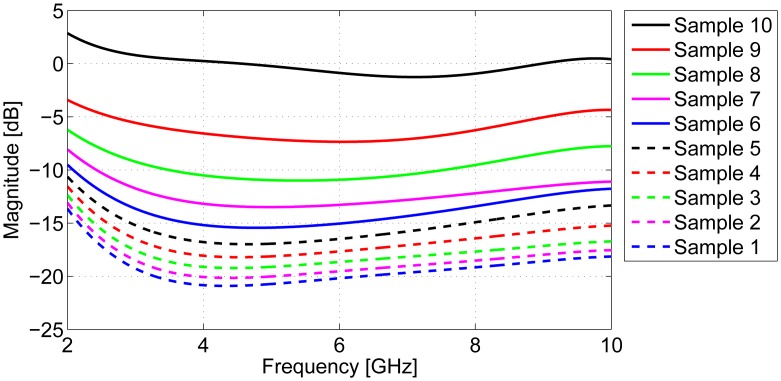
Magnitudes of the computed antenna responses for each tissue group.

The magnitudes of the antenna responses are shown in Figure 5, illustrating that the antenna response is highly influenced by the DPs of the breast tissue, with lossy tissues (Sample 1) requiring more compensation than low-property tissues (Sample 10).

In order to use the correct antenna response when analyzing measured data, the closest tissue group for the OUT must be identified. To do this, the theoretical path loss for a specific distance was found for each tissue group (Equation (4)) and then convolved with the corresponding antenna response found in the previous section:

(6) $$S_{21,\text{sample}i}\left(f,R\right)=PL_{\text{theo}.,\text{sample}i}\left(f,R\right)\cdot G_{\text{samplei}}\left(f\right)$$

The measured data were then compared to the theoretical results to identify the closest tissue sample and, therefore, the corresponding antenna response. The closest antenna response was then used to deconvolve the measured data and isolate the breast tissue path loss:

(7) $$PL\left(f,R\right)=\frac{S_{21,measured}\left(f\right)}{G_{\text{sample}i}\left(f\right)}$$

#### 2.2.3. Average Dielectric Property Estimation

With the isolated tissue response, it was possible to estimate the average DPs. To begin, a single relaxation Debye model was assumed (Equation (1)). Based on [11], a single pole was sufficient to represent breast tissues over the frequency range of interest. Using an iterative approach, the various Debye parameters were estimated (*i.e.*, ϵ_∞_, ϵ*_S_, τ* and *σ_S_*). This method began with a range of values for each Debye parameter. To provide an initial guess, these estimates were centered on the properties of the tissue group that were estimated in Section 2.2.2 and extended to the properties of the tissue group above and the tissue group below the initial guess. Every combination of parameters was then found. For example, if each of the four Debye parameters had a range of 10 values, this means that 10^4^ combinations were created. For each combination of parameters, the complex permittivity was found with Equation (1), and then, the path loss was found with Equation (4). The average relative error between this path loss and the measured results was calculated. For each iteration, the best combination was found by finding the minimum relative error. These results formed the basis for the next iteration. For this technique, we found that 10 values for each parameter and five iterations were ideal. The final result was a dispersive average DP estimation for the OUT.

#### 2.2.4. Processing Time

The signal-processing technique described above can be done quickly to provide imaging algorithms with *a priori* average dielectric property information. The data were analyzed using a typical desktop computer (XPS 8500 with a 3.4-GHz Intel Core i7-3770 CPU and 16 GB RAM, Dell Inc., Round Rock, TX, USA), and the signal-processing code was written in MATLAB (MathWorks Inc., Natick, MA, USA). The code was written to generate many figures to ensure each step was done correctly; however, assuming four measurements were taken, results could be found in less than 30 s. For the time-gating step, the dominant factor was the user input to identify the number and location of peaks, as well as plotting the results. The processing time for the time-gating step was on the order of a few milliseconds. Similarly, the processing time for the antenna compensation step was a few milliseconds, but no input was required. The average dielectric property estimation step took the longest; however, it was not unreasonable and typically took 3 s per measurement.

## 3. Validation

First, the techniques presented in the previous section are tested on simulations of a material with properties different from those used to calculate the antenna compensation factor. Next, the changes in the antenna compensation with differences in the properties of skin and the separation distances between the sensors are investigated. Finally, results obtained with simulations and measurements are compared to ensure that the antenna compensation factor obtained with simulations is applicable to measured data.

### 3.1. Simulated Data

**Figure 6.**
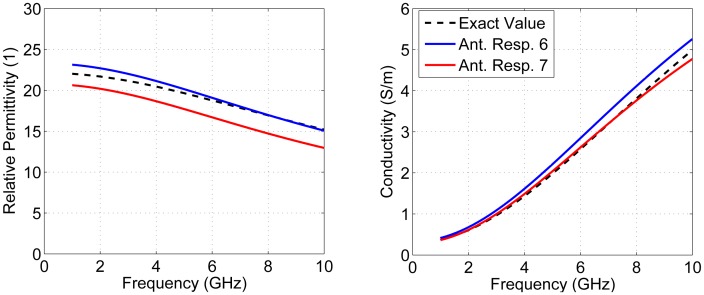
Average dielectric property (DP) estimate for a simulated tissue with DPs between Samples 6 and 7.

To test the average DP estimation technique, a simulation was created with different properties than what was used to calculate the antenna compensation. The simulation model shown in Figure 4 was used, with the DPs of the homogeneous tissue layer changed to be between Samples 6 and 7 (ϵ_∞_ = 4.96, ϵ*_S_* = 22.14, *τ* = 13.10 ps and *σ_S_* = 0.2995 Ω ^−1^m^−1^). The average DPs were estimated twice: once using the antenna response corresponding to Sample 6 and once using Sample 7. The results are shown in Figure 6. The average DP estimate for both cases was reasonably close to the actual value. The error seen in Figure 6 is due to the fact that no antenna compensation factor was optimized specifically for this tissue. This error could be reduced by increasing the number of tissue samples used to find the antenna response in Section 2.2.2 or by interpolating the antenna response for tissues that fall between two groups.

For radar-based techniques, both estimates shown in Figure 6 provide an adequate estimation of the phase velocity. For example, consider a target located 5 cm from the aperture of an antenna in a radar-based system. If we know the time-of-flight (*τ*) to and from the target, Equation (8) can be used to determine the distance (*d*):

(8) $$d=\frac{c\cdot \tau }{2\sqrt{\in_{r}}}$$

where *c* is the speed of light in a vacuum and ϵ*_r_* is the average permittivity. For the tissue shown in Figure 6, the average permittivity from 2 to 8 GHz is 19.3, which means that the time-of-flight would be 1.46 ns. The average permittivities using the estimated results in Figure 6 are 19.8 and 17.5 using antenna Responses 6 and 7, respectively. Using Equation (8) and the same time-of-flight, these results estimate distances of 4.9 cm and 5.3 cm, respectively. Compared to guessing a permittivity of nine, which would results in a distance of 7.3 cm, these DP estimations would both substantially improve imaging results.

### 3.2. Antenna Response Simulations

**Figure 7.**
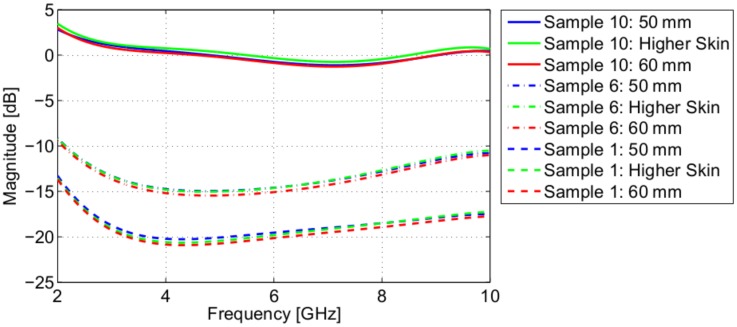
Antenna response variation due to different skin properties and different separation distances.

When measuring participant breast tissue, the separation distance and the skin properties may be different than what was used in the simulation used to obtain the antenna compensation. Simulations were therefore created to test the effect of different skin properties and different separation distances. First, a simulation was created with skin properties that were 10% higher than what was reported in [10]. Second, a simulation was created with 46 mm of homogeneous breast tissue (50 mm of separation between the antennas). The simulated transmission coefficient was then used to find the antenna response (*G*(*f*)). The results for tissue Samples 1, 6 and 10 are shown in Figure 7. Different skin properties and a different separation distance resulted in a very small change in the magnitude of the antenna response. This could result in a small change in the average DP estimate; however, relative to the magnitude of typical transmission coefficients (e.g., Figure 3), these changes are very small. These results suggest that the assumption that *G*(*f*) does not depend on separation between sensors is reasonable, as well as the robustness to differences in the properties of the skin.

### 3.3. Simulations versus Measurements

The antenna compensation was estimated using simulations. To ensure that these simulations are representative of the real system, measurements were compared with these simulations, and the error was assessed.

**Figure 8.**
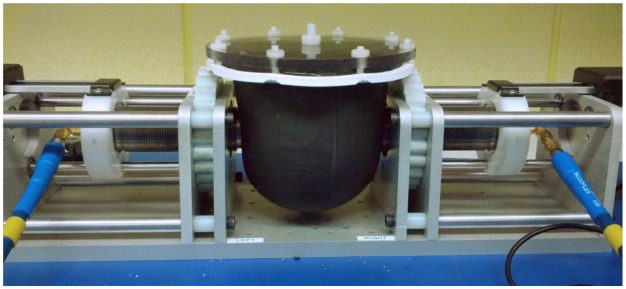
Transmission system with the breast phantom present.

To generate measurement data, a breast phantom [26] was used as the OUT. The skin layer for this phantom was 2 mm thick, 10 cm wide and 9 cm tall. It was composed of 20% graphite and 80% urethane, by weight. This provided an average relative permittivity of 10. The phantom is shown in Figure 8. The measurement methodology described in Section 2.1 was used: once with canola oil inside the skin layer and once with glycerin inside the skin layer.

Simulation results were generated using the model shown in Figure 4. For the DPs, a dielectric probe [27] was used to measure the physical OUT, and these DPs were uploaded into FDTD simulation software (SEMCAD, Speag, Zurich CH) and used during the simulation.

**Figure 9.**
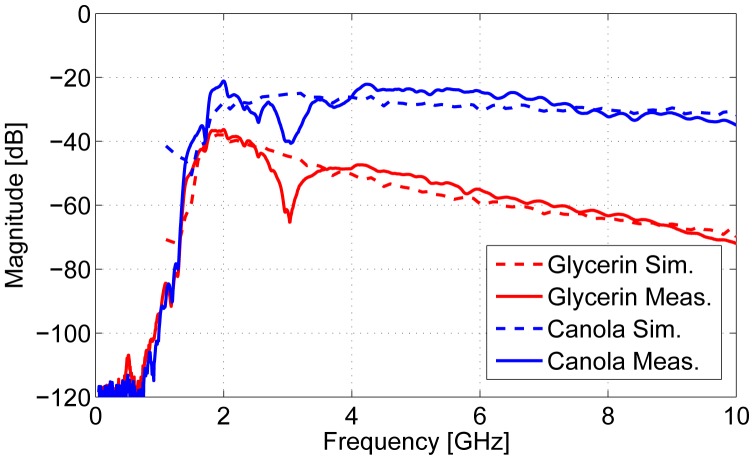
Comparison between simulated and measured results using canola oil and glycerin.

The comparison between simulated and measured data is shown in Figure 9. These results show that the simulations provide an adequate approximation of the physical system. The measured results feature a resonance at 3 GHz. This is likely due to a reflection related to the skin layer. The simulated results do not feature this resonance, because a simple planar slab model was used as the OUT.

**Figure 10.**
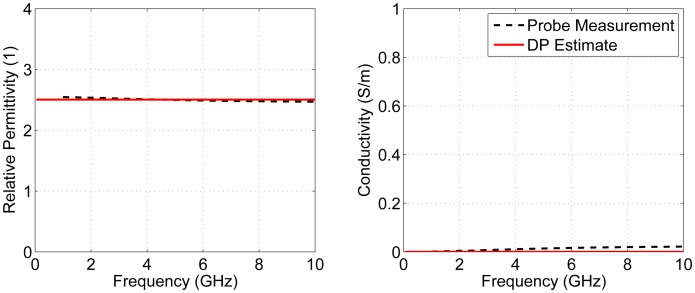
Estimated average DPs for canola oil using the measured results.

Next, the accuracy of the average DP estimation technique was assessed. For the breast model filled with canola oil, six transmission measurements were completed using the system shown in Figure 8. After each measurement, the phantom was removed, rotated and replaced. Since the properties of canola oil are very low, a new antenna response was found with the technique described in Section 2.2.2 using the properties of canola oil. Figure 10 shows the estimated average DPs for canola oil. The estimate was very close to the value measured with the dielectric probe with average absolute errors of 0.02 and 0.01 S/m for permittivity and conductivity, respectively. This was a very simple case, because canola oil has very low properties and is not very dispersive.

**Figure 11.**
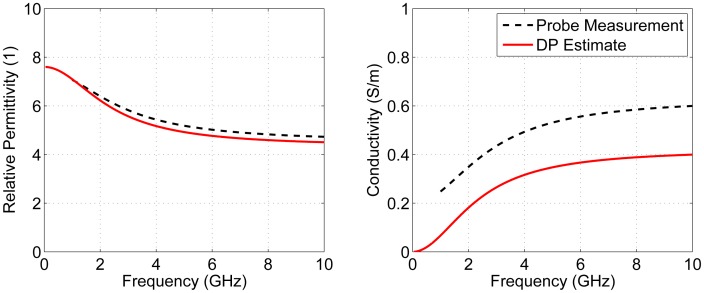
Estimated average DPs for glycerin using measured results.

This testing procedure was then repeated with glycerin placed inside the breast. Glycerin has much higher permittivity and conductivity than canola oil. The results are shown in Figure 11. The average DP estimate for glycerin was also reasonably close to the value measured with the dielectric probe; however, it was slightly underestimated with average absolute errors of 0.23 and 0.19 S/m for permittivity and conductivity, respectively. This is due to the slight discrepancy seen in Figure 9, where measured data seem to have less attenuation than simulated data.

The estimates of the average properties of materials filling the breast phantoms demonstrates the potential of the technique. The simulations are in good agreement with the measurements, resulting in reasonably accurate property estimates.

## 4. Application to Patient Data

To date, six patients have been scanned with the 5 × 5 array system. Very lossy tissues, heterogeneous distributions and/or multipath were noted for several patients. Below, the data from the most recently scanned patient are analyzed.

**Figure 12.**
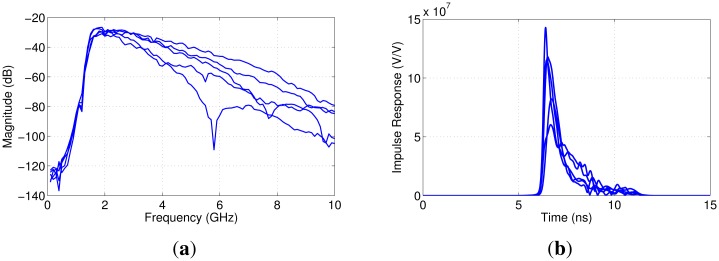
Measured transmission results between five sensor pairs for a recent participant. (**a**) Frequency domain; (**b**) time domain.

To begin, the transmission coefficient was measured between each pair of antennas for a total of five measurements (only antennas that were directly aligned were used from this analysis, and minimal coupling between adjacent sensors was noted). The transmission coefficient and the time domain impulse response for each of the sensor pairs are shown in Figure 12. The transmission coefficient in the frequency domain shows signs of multipath for several measurements and significant variation in magnitude between the measured signals. The Nahanni sensors operate from 1.8 to 12 GHz, which explains the low-frequency behavior. The time domain response also shows variation in terms of amplitude, and the peaks are relatively broad, which suggests multipath and dispersive tissues.

**Figure 13.**
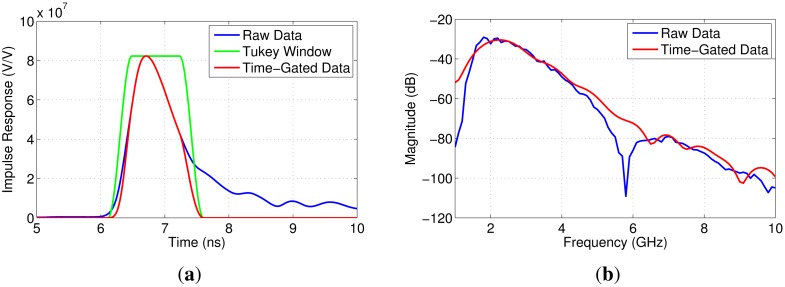
The results of time-gating for one of the five transmission measurements. (**a**) Time domain; (**b**) frequency domain.

**Figure 14.**
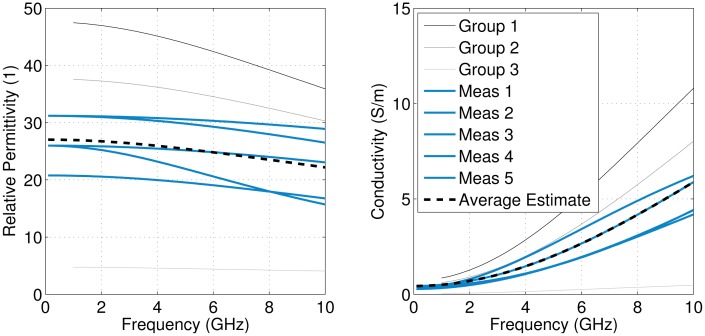
Estimated average DPs for results taken from a recent patient. The DPs of different breast tissue groups is included for comparison [11].

Time-gating was performed to isolate the dominant peak for each of the five measurements. Figure 13 shows the results of time-gating for a measurement that exhibited strong multipath characteristics. Antenna compensation was then applied, and the data from 2 to 10 GHz were used to estimate the average DPs of the breast tissue. The results are shown in Figure 14. Since variation was observed in the transmission coefficients, variation in the average DP estimate is expected. All of the estimates are reasonable, and the antennas are likely interrogating regions that contain different amounts of adipose tissue and glandular tissue, as expected from the tissue structure in the breast. Imaging results from another modality (X-ray or magnetic resonance imaging) would assist in interpreting these results, as information on the structure of the tissue would be available. Microwave tomography could also be used to gather structural information; however, the 5 × 5 transmission system used here was not designed for imaging, and applying inverse scattering techniques would be very challenging. Overall, the average properties are in the expected range for breast tissues, demonstrating the potential of this technique.

## 5. Conclusions

A new signal-processing approach to analyze transmission measurements of breast tissue has been developed. This technique is resistant to multipath and provides an estimation of complex permittivity over a range of frequencies. For this approach, two sensors are placed on either side of the breast, and a vector network analyzer is used to measure the transmission coefficient from 50 MHz to 10 GHz. A signal-processing procedure is then used to estimate the average dielectric properties. This involves three primary steps. Firstly, time-gating is used to eliminate multipath artifacts from the transmission measurements. For this, a Tukey window is used to select the dominant peak in the time domain. Secondly, an antenna compensation step is applied. The antennas' responses were simulated using complex models and FDTD software for a variety of tissue types. The correct tissue type is then identified through an iterative process, and the antennas' response is removed from the transmission measurement. Finally, the average dielectric properties are estimated through an iterative process that provides the parameters for a dielectric relaxation model. This technique was validated with simulated measurement data to assess the approach's sensitivity to different breast properties, the assumptions made during the antenna compensation step and the applicability to measurement data. The latter was tested further by analyzing transmission data taken from breast phantom measurements. Finally, this technique was also tested with transmission measurements taken from a recent patient study. Although the correct properties are not known, the technique again provided reasonable results. Further testing with additional patient data and comparison of results with clinical breast images are planned.

## Figures and Tables

**Table 1.** Dielectric properties of the linear range of tissues used in the simulations.

**Sample Number**	**ϵ_∞_**	**ϵ***_S_*	***τ* (ps)**	***σ****_S_* **(S/m (S = Ω ^−1^))**
1	7.82	49.30	10.66	0.713
2	7.30	44.36	11.10	0.638
3	6.78	39.42	11.55	0.563
4	6.26	34.48	11.99	0.487
5	5.74	29.54	12.43	0.412
6	5.22	24.61	12.88	0.337
7	4.70	19.67	13.32	0.262
8	4.18	14.73	13.79	0.186
9	3.66	9.79	14.21	0.111
10	3.14	4.85	14.65	0.036

**Figure A1.**
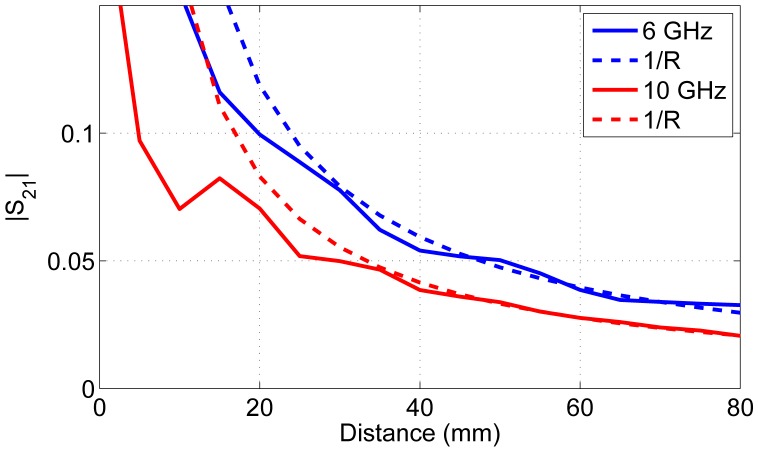
Power roll-off as a function of distance to depict the characteristics of far-field radiation.

## A. Appendix: Far-Field Assumptions for Path Loss

Equations (2)–(4) assume that the antennas are operating in the far-field. In the far-field, the power transmission ratio rolls off as 1/*r*^2^; therefore, the magnitude of *S*_21_ should roll off as 1/*r*.

To investigate this, measurements were completed in free-space using the system shown in Figure 1. The first measurement was taken with no separation between the antennas. This distance was then increased in 5-mm increments to 80 mm. The results from two frequencies are shown in Figure A1. For each dataset, a 1/*r* curve was fit to the data. These results show that the far-field assumption is valid for distances greater than approximately 4 cm in free-space. For breast tissue, this distance should be even shorter, because the wavelength will be shorter, as well.
